# Survivorship issues in long‐term survivors of locally recurrent rectal cancer: A qualitative study

**DOI:** 10.1111/codi.70051

**Published:** 2025-03-20

**Authors:** Niamh McKigney, Sophia Waldenstedt, Elisabeth Gonzalez, Jan M. van Rees, Henriette Vind Thaysen, Eva Angenete, Galina Velikova, Julia M. Brown, Deena P. Harji, LRRC‐QoL Collaborators, Henrik Kidmose Christensen, Joost Rothbarth, Cornelis Verhoef, Elliott Gee, Kaitlyn Ulmer, Ahmer Karimuddin, Claire Taylor, Laura Gould, Elaine Burns, John T. Jenkins, Chloe Hardy, Christopher Mann, Kirsten Boyle, David McArthur, Mit Dattani, Cath Moriarty, Peter Sagar, Tamara Glyn, Frank Frizelle, Helen Mohan, Satish Warrier, Alexander Heriot, Tracy Fitzsimmons, Tarik Sammour

**Affiliations:** ^1^ Clinical Trials Research Unit, Leeds Institute of Clinical Trials Research University of Leeds Leeds UK; ^2^ Department of Surgery, SSORG‐Scandinavian Surgical Outcomes Research Group Institute of Clinical Sciences, Sahlgrenska Academy, University of Gothenburg Gothenburg Sweden; ^3^ Region Västra Götaland Sahlgrenska University Hospital, Transplant Centre Gothenburg Sweden; ^4^ Department of Surgical Oncology and Gastrointestinal Surgery Erasmus MC Cancer Institute, University Hospital Rotterdam Rotterdam The Netherlands; ^5^ Department of Surgery Aarhus University Hospital Aarhus Denmark; ^6^ Leeds Institute of Medical Research at St James's University of Leeds, St James's University Hospital Leeds UK; ^7^ Leeds Cancer Centre, Leeds Teaching Hospitals NHS Trust St James's University Hospital Leeds UK; ^8^ Department of Colorectal Surgery Manchester University NHS Foundation Trust Manchester UK

**Keywords:** locally recurrent rectal cancer, long‐term survivorship, unmet needs

## Abstract

**Aim:**

There are increasing numbers of long‐term survivors following curative treatment for locally recurrent rectal cancer (LRRC); however, their experiences remain relatively underreported. The aim of this qualitative study was to identify the long‐term survivorship issues relevant to these patients.

**Method:**

Adults who remained disease free >3 years following treatment for LRRC were invited to participate in an international multicentre study. Semistructured qualitative interviews were conducted either in person, via telephone or via Microsoft Teams and were analysed using a framework method of thematic analysis.

**Results:**

A total of 26 participants were recruited from 11 sites in seven countries. Sixteen (61.5%) participants were male, the median age was 70.5 (33.0–85.0) years, participants were a median of 5.0 (3.0–17.0) years posttreatment, most had undergone surgery for LRRC (*n* = 24, 92.3%), two participants received neoadjuvant chemo/radiation for LRRC with a complete response. Eight major survivorship themes were identified: (1) experience of long‐term follow‐up care, (2) unmet needs and areas for improvement, (3) long‐term physical effects of cancer and treatment, (4) living with a stoma, urostomy or other urinary device, (5) long‐term psychological impact, (6) impact on sexual function and intimate relationships, (7) impact on daily life and (8) feelings surrounding life now, adapting and the future.

**Conclusion:**

Participants experienced a wide range of long‐term survivorship issues, reflecting the complexity of both LRRC and its treatment. Despite this, most had adapted well. Further work regarding survivorship care in LRRC is required to address the unmet needs and issues highlighted in this study, such as support regarding sexual function.

**What does this paper add to the literature?**

This study represents an important step in understanding the lived experiences of long‐term survivors of locally recurrent rectal cancer. A wide range of survivorship issues were identified, with a pervasive and sometimes burdensome impact on participants' lives. The unmet needs identified represent an important area for future work.

## INTRODUCTION

The multidisciplinary management of locally recurrent rectal cancer (LRRC) has evolved significantly over the past three decades and curative approaches involving radical surgery, often in combination with neoadjuvant treatment, are routinely delivered at specialist centres [1, 2, 3, 4, 5, 6, 7, 8, 9]. The anatomical boundaries for resection have been expanded through ultraradical approaches [10], such as extended lateral pelvic sidewall excision (ELSiE) and high sacrectomy [11, 12, 13], with reported 5‐year survival rates of 34.5%–44.6% following complete (R0) resection [2, 6]. However, these procedures are associated with high postoperative morbidity, with reported rates of up to 60% [14, 15, 16].

As curative treatment strategies for LRRC become more routine and acceptable, along with the continual improvement in outcomes and survival, due attention is now being focused on understanding the longer‐term consequences of pursuing curative surgery. The long‐term postoperative physical effects are sparsely reported, but include impaired mobility and foot drop following sciatic nerve resection [17], empty pelvis syndrome [18] and urological complications [18].

Focusing on cancer survivorship in this cohort of patients is pertinent given the growing number of people living with and beyond cancer [19].

Survivorship issues address the range of problems that are relevant to cancer survivors, including late effects of treatment and health‐related quality of life (HRQoL) [20]. Identifying survivorship issues relevant to this specific group of patients could be used to inform shared decision‐making processes regarding treatment and to develop survivorship care models. To date, there is limited evidence regarding survivorship in LRRC [21], particularly in longer‐term survivors. The overall aim of this study was to identify the survivorship issues relevant to adults who have been treated for LRRC and remained disease free for 3 years or longer.

## METHOD

A multicentre, international qualitative study was undertaken between November 2020 and July 2023. Eleven centres were recruited internationally, including centres from the UK, Australia, Sweden, New Zealand, Denmark, Canada and the Netherlands. The study was approved by the West of Scotland Research Ethics Committee (ref. 20/WS/0116) with additional ethical approvals at each participating international centre. The study is reported in keeping with Standards for Reporting Qualitative Research (SRQR) [22].

### Eligibility criteria and recruitment

Individuals were invited to participate in the study if they were treated for LRRC more than 3 years ago and remained disease‐free and were able to provide informed written consent. Potential participants were excluded if they had been diagnosed with distant metastases or locally re‐recurrent rectal cancer, or if they had a history of cognitive impairment. A purposive recruitment strategy was used to recruit participants reflecting the diversity of LRRC survivors, aiming to recruit a minimum of four participants per key factor, including sex, location of LRRC and neoadjuvant treatment (this is further detailed in the Supporting Information Figure S1). Participants were identified by clinical teams at participating centres from existing local registries and approached either via telephone, via post or during follow‐up clinic appointments, and provided with a patient information leaflet, a consent form and a demographics form to complete.

### Data collection

Demographic data were collected using a self‐complete form, this included data on age, sex, ethnicity, marital status, education and employment status. Clinical data were collected, including date of diagnosis with LRRC, mode of detection, pattern of LRRC, preoperative treatment, operation performed, date of surgery, margin status and postoperative treatment.

Individual qualitative, in‐depth, semistructured interviews were undertaken using an interview topic guide (see Supporting Information). The Locally Recurrent Rectal Cancer—Quality of Life (LRRC‐QoL) conceptual framework was used to inform the topic guide with additional questions to explore participants' experiences [23]. Interviews were facilitated by researchers who were native speakers of the same language as the participant: NM (English), SW and EG (Swedish), HvT (Danish) and JvR (Dutch). All interview facilitators had either received training in qualitative methodology or were experienced qualitative researchers. The characteristics of the researchers and how they may have influenced the research are detailed in the Supporting Information. Interviews were undertaken either via telephone, Microsoft Teams or in person; patients were either interviewed from their own home via telephone or Microsoft Teams or in a clinical setting to enable face‐to‐face interviews. Many of the interviews took place during the COVID‐19 pandemic or across different countries, meaning there was a predominance of remote methods. Each interview was audiorecorded and transcribed verbatim. A reflective log was maintained throughout the delivery of the study to critically evaluate the researcher's role within the research [24].

### Data analysis

A descriptive analysis was used for the demographic and clinical data, using SPSS Statistics for Mac, version 26 (IBM Corp., Armonk, NY, USA). A framework method was used for the qualitative analysis [25, 26, 27]. Transcripts were analysed sequentially following one to three interviews using NVivo 12 software or an Excel spreadsheet by NM and SW. Regular meetings were held to ensure agreement in coding and to update the working analytical framework. A framework method of thematic analysis was chosen to enable collaborative working and the coordination of the study at multiple international sites. A combined inductive–deductive approach was used. Coding was not predetermined prior to commencing the analysis; however, the identification of codes and themes was informed by the development of the LRRC‐QoL conceptual framework. During the development of the framework, a subset of transcripts was reviewed by a second researcher. Recruitment to the study continued until no new emerging themes were identified and thematic saturation was reached [28]. In the context of this study, the approach was taken that no new themes were identified following two sequential sets of three interviews. Transcripts in Swedish were analysed in their original form, with coding and quotations translated into English. Transcripts in Dutch and Danish were translated into English for analysis and discussed with the researcher who undertook each interview to ensure conceptual equivalence.

## RESULTS

Thirty‐one participants were recruited to the study; five were excluded following interviews as they were found not to meet the eligibility criteria due to having developed re‐recurrence or metastatic disease. A total of 26 participants were interviewed and included in the qualitative analysis.

### Clinical and demographic characteristics

The clinical and demographic characteristics are detailed in Table 1. Sixteen (61.5%) participants were male and most were of White ethnicity (*n* = 16, 61.5%). The median time interval since either diagnosis or surgical treatment for LRRC was 5.0 years (range 3.0–17.0 years). The majority of participants had undergone surgery for LRRC (*n* = 24, 92.3%). Two participants received neoadjuvant chemo/radiation more than 3 years ago, achieved a complete clinical response and remained disease‐free following biopsy‐proven LRRC.

**TABLE 1:** Demographic and clinical characteristics.

Characteristics	Participants
Median age (range) (years)	70.5 (33–85)
No. of participants recruited per country	
United Kingdom	11 (42.3%)
Sweden	7 (26.9%)
New Zealand	1 (3.8%)
Denmark	1 (3.8%)
Canada	3 (11.5%)
The Netherlands	1 (3.8%)
Australia	2 (7.7%)
Interview setting	
Face to face	9 (34.6%)
Telephone	16 (61.5%)
Video call	1 (3.8%)
Sex	
Male	16 (61.5%)
Female	10 (38.5%)
Ethnicity	
White	16 (61.5%)
Black	1 (3.8%)
Asian	1 (3.8%)
Unknown or not reported	8 (30.8%)
Marital status	
Married	15 (57.7%)
Living with partner	1 (3.8%)
Divorced	1 (3.8%)
Single	3 (11.5%)
Unknown	6 (23.1%)
Education status	
Secondary school	5 (19.2%)
College	8 (30.8%)
University	3 (11.5%)
Other	1 (3.8%)
Unknown	9 (34.6%)
Employment status	
Self‐employed	1 (3.8%)
Full‐time employment	1 (3.8%)
Part‐time employment	2 (7.7%)
Retired	15 (57.7%)
Other	1 (3.8%)
Unknown	6 (23.1%)
Median time since LRRC (range) (years)	5.00 (3.00–17.00)
Mode of detection	
Symptomatic	9 (34.6%)
Surveillance	10 (38.4%)
Unknown	7 (26.9%)
Pattern of LRRC	
Anterior	6 (23.1%)
Central	5 (19.2%)
Lateral	8 (30.8%)
Posterior	3 (11.5%)
Unknown	4 (15.4%)
Preoperative treatment	
None	10 (38.4%)
Short‐course radiotherapy	2 (7.7%)
Long‐course chemoradiotherapy	8 (30.8%)
Long‐course chemoradiotherapy and chemotherapy	1 (3.8%)
Chemotherapy	2 (7.7%)
Unknown	3 (11.5%)
Operation performed for LRRC	
APE	5 (19.2%)
APE, hysterectomy, salpingo‐oophrectomy and resection of vagina	1 (3.8%)
APE and resection and reconstruction of ureter	1 (3.8%)
APE, S1/2 sacrectomy, ureteric catheters and VRAM flap	1 (3.8%)
Cystectomy with Bricker and resection of small bowel	1 (3.8%)
ELAPE	1 (3.8%)
ELAPE, right pelvic side wall resection and presacral fascia, reversal of ileostomy and formation of end colostomy	1 (3.8%)
ELAPE, coccygectomy, prostatectomy, vesiculectomy, unilateral IGAP flap, distal ileal resection	1 (3.8%)
Infralevator total pelvic exenteration, distal sacrectomy, reversal of loop ileostomy, end colostomy, ileal conduit and left IGAP flap	1 (3.8%)
Low Hartmann's procedure	1 (3.8%)
Pelvic exenteration: cystectomy, resection of ureter with Bricker, resection of vagina, neorectum left in situ	1 (3.8%)
Posterior exenteration	1 (3.8%)
Posterior exenteration, S3 sacrectomy, reimplantation of left ureter, excision of seminal vesicles and end colostomy	1 (3.8%)
Rectal resection, ileocaecal resection and resection of ureter, end colostomy	1 (3.8%)
Redo anterior resection and left ELSiE	1 (3.8%)
Right ELSiE and parastomal hernia repair	1 (3.8%)
Total right pelvic sidewall excision with right salpingo‐oophrectomy	1 (3.8%)
None, complete response of biopsy confirmed LRRC to chemotherapy	1 (3.8%)
None, complete response of biopsy confirmed LRRC to total neoadjuvant therapy	1 (3.8%)
Margin status	
R0	17 (65.4%)
R1	3 (11.5%)
R2	1 (3.8%)
Not applicable	2 (7.7%)
Unknown	3 (11.5%)
Postoperative treatment	
None	20 (76.9%)
Chemotherapy	2 (7.7%)
Unknown	4 (15.4%)

Abbreviations: APE, abdominoperineal excision; ELAPE, extra‐levator abdominoperineal excision; ELSiE, extended lateral pelvic sidewall excision; IGAP, inferior gluteal artery perforator; LRRC, locally recurrent rectal cancer; VRAM, vertical rectus adbominis myocutaneous.

### Survivorship issues and themes identified

**FIGURE 1:**
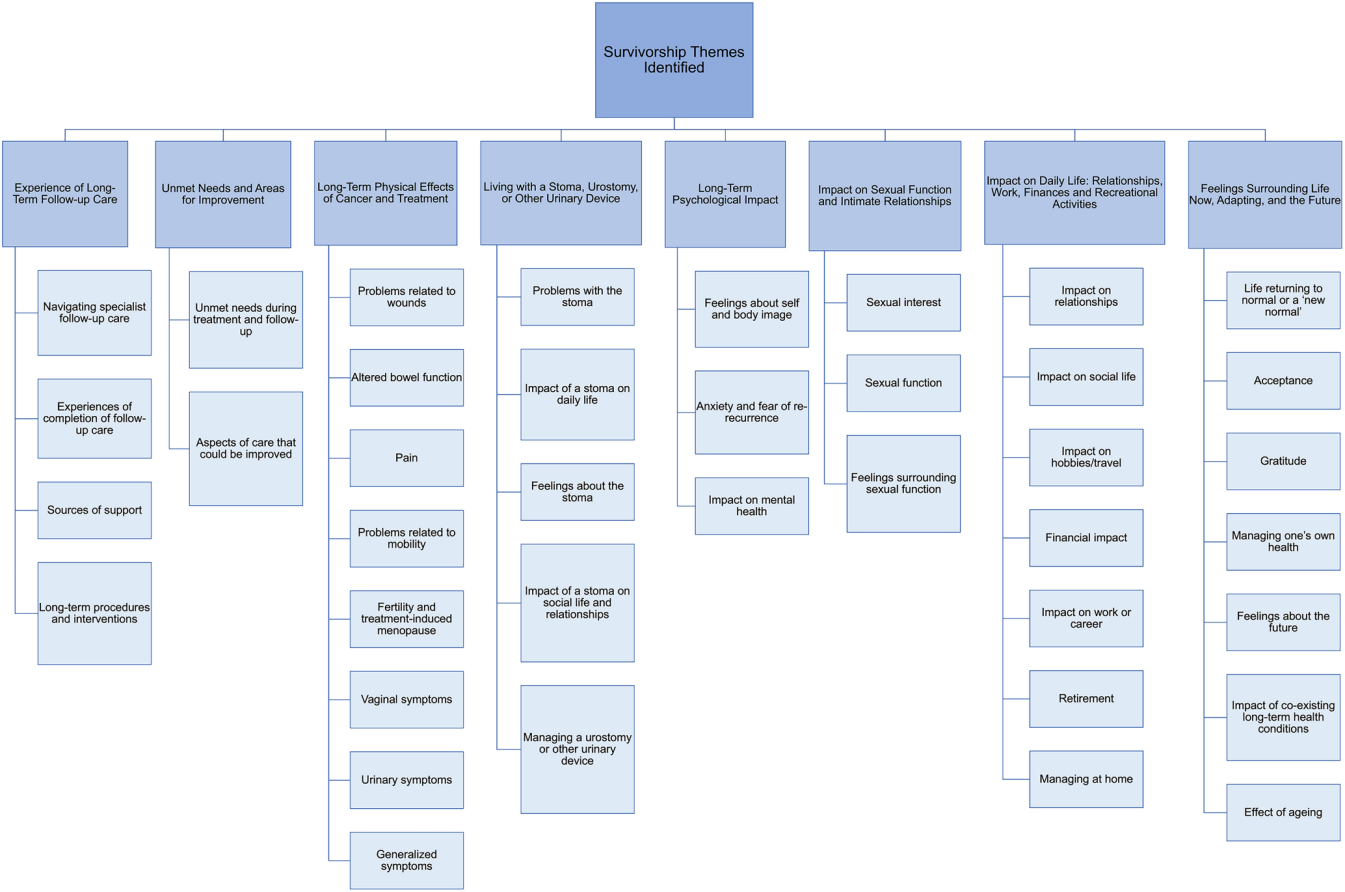
Survivorship themes identified.

Eight major survivorship themes were identified (Figure 1) and one theme related to reflections on adjusting to life following diagnosis and during treatment. The survivorship themes identified were: (1) experience of long‐term follow‐up care, (2) unmet needs and areas for improvement, (3) long‐term physical effects of cancer and treatment, (4) living with a stoma, urostomy or other urinary device, (5) long‐term psychological impact, (6) impact on sexual function and intimate relationships, (7) impact on daily life: relationships, work, finances and recreational activities and (8) feelings surrounding life now, adapting and the future. Tables 2 and 3 provide illustrative quotations for each theme.

**TABLE 2:** Survivorship themes identified with illustrative quotations.

Themes identified	Quotations
Experiences of long‐term follow‐up care	
Navigating specialist follow‐up care	‘The only thing I dread are these recurring trips to (the specialist hospital), using an expensive parking and finding my way in busy city traffic. That's a bit of a bother, but I'm happy to go there.’ ‘So, the thing that was really clear about all of the people that were involved and became my medical team, there was no holistic approach, it was all, everything was very siloed.’
Experiences of completion of follow‐up care	‘Now if I had my way, if I had my way, this is probably slightly paranoid, I would still carry on having the scans, because as far as I'm concerned you can never be too careful.’
Sources of support	‘My doctor is great, but I always feel rushed around them, with the waiting room full as it is. He's so busy, that I tell myself to hurry up, so you forget half of what you wanted to say/ask. With her however (specialist nurse), I'm at ease, taking my time. I can even email her with questions afterwards. I was really happy with this combined approach of physician and nurse.’
Long‐term procedures and interventions	‘I had to have an emergency op because my small intestine had perforated.’ ‘I had abdominal reconstruction, after the first surgery I had problems with hernias and they became very large and so I've had to have quite a few operations.’
Unmet needs and areas for improvement	
Unmet needs during treatment and follow‐up	‘In terms of sort of like, you know, the physical aftereffects of surgery on my libido and things like that, that's just never been talked about actually really, and maybe that's remiss of me not to be more upfront and ask what they could do to help. But no, there's been very little aftercare.’
Aspects of care that could be improved	‘I think sort of greater communication between everybody would really help that just sort of being able to feel engaged with your treatment plan. You know, without having to go off and do Dr Google (laughter) which often can be quite negative.’
Long‐term physical effects of cancer and treatment	
Problems related to wounds, including abdominal wounds, perineal wound, rectal stump, and myocutaneous flaps	‘I mean it discharges all the time, you know, if I don't wear underpants with pads on the inside, my bedcovers are covered in it in the morning, you know I'm forever washing them.’
Altered bowel function	‘Things can move a lot quicker. Erm, but you know, when it first started, just after the surgery, well it was more after the radiotherapy I suppose, I thought that I'd never be able to go on a long day hike or go camping or things like that, things that I really love. So, that's improved so much really and I am pretty free. I don't feel that it really stops me, I mean, if my movements are a lot quicker than I want them to be, I can just take some Immodium, that's pretty manageable.’
Pain	‘I've got pain from my buttock going down my right leg down to my foot. It's like a burning pain, as if I've got some nerve damage from the operation.’
Problems related to mobility	‘So it's kind of like restricted movement, I can't, if I'm standing up, I can't stand on that leg, because I can't push down with my toes to balance’, ‘it's my foot. I can't, my left foot, I can't, I can't move it on it's own, it's dropped all the time, so I have a, like a support that just keeps the foot at right angles from the leg.’
Fertility and treatment‐induced menopause	‘erm… I think the biggest impact was, I haven't even talked about this yet, was when I started having radiation, erm, I think it was within 2 weeks, I went from having a period to never having a period again’, ‘I was running so hot, having the hot flashes. Again, which seems so like, you know when you think about it, of course I was having hot flashes when you've had radiation and you don't have …’
Vaginal symptoms	‘Initially, I was very worried about that. “How's that possible, I can't be menstruating (after the surgery), so what can it be?” I've had frequent checks with the gynaecologist, including a pelvic exam, and I had oxygen therapy, but nothing has changed. It's still the same, even now. My gynaecologist has tested and examined me for it since 2019. Nothing has changed though, it keeps coming, so I just accepted it.’
Urinary symptoms	‘With a Tena nappy. I make sure I've got one in the car and one with me, wherever I am. Erm and make sure I'm wearing a skirt, or a dress, so that I can quickly tear the sides and put it on. So that I can actually go, because several times I've been caught out.’
Generalized symptoms	‘Generally, I suppose, since the operation I think I've probably felt more tired, you know towards the end of the day.’
Living with a stoma, urostomy, or other urinary device	
Problems with the stoma	‘The stoma size has changed over the years and so I have had to go see stoma nurses to help re‐fit things if I was experiencing leakages, erm … or leakage or just like, different kinds of friction or whatever.’
Impact of a stoma on daily life	‘I try not to be too far away from a toilet because it's something that you have to manage quite regularly and unfortunately it does dominate … it can dominate a large part of the day.’
Feelings about the stoma	‘I'm really happy with my colostomy … my quality of life is a lot better with that than with the TME [total mesorectal excision] procedure. If I had to make a long trip for my work, I left the house with diapers on.’
Impact of a stoma on social life and relationships	‘Well, I think probably just, I'm always thinking about “is my bag showing?” I don't mean hanging out of my clothes, just the actual shape, does it show. If I'm in a close social event, with people close by, then I think about the farting part of it. So, I'm sort of conscious.’
Managing a urostomy or other urinary devices	‘I don't wear a leg bag anymore but I used to wear it … I clicked the valve once when I was talking to people and once the valve came off when I was going round the supermarket.’
Long‐term psychological impact	
Feelings about self and body image	‘Erm, well, I haven't had a physical relationship with anyone for years now but I wouldn't have felt confident to anyway’, ‘You know, the sight of the hernia and all the bits and pieces that are missing now (laughs), I wouldn't have been very body‐confident.’
Anxiety and fear of re‐recurrence	‘But even now, I might be a little bit paranoid, but any little thing that doesn't feel normal, you start thinking the worst and it's been even worse these past 12 months, not being able to talk to anyone.’
Negative effects on mental health	‘Because pain makes you feel grouchy all day or very quiet and people misunderstand that my grouchiness is not because of them, it's just because I'm in pain, or my quietness is not because of them. I'm not being arrogant or rude, it's just because I'm in pain.’
Positive effects on mental health	‘Just having gone through it twice, you know, it gives you a totally different outlook on life and it makes you realize how precious life is.’
Impact on sexual function and intimate relationships	
Sexual interest	‘We still do it, but with a lot less penetration. It's not always nice. Nor do I know why I'll “allow” it sometimes, and not at other times. So we can do it, and it “works”, but because of this painful moment, I sometimes decline.’
Sexual function	‘I did, through surgery, sustain a little bit of nerve damage to my vulva and around my clitoris which was slightly disappointing in that regard, so I don't have as much sensation down there as I used to.’ ‘Yes, there's no sex now. There's nothing happening down there at all [erectile dysfunction].’
Feelings surrounding sexual function	‘My self‐image has changed a lot though: I hate the sight of my vagina with that flap that was folded inwards to close my anus and repair the backwall. So there are indeed positions that are a no‐go for me; I really don't want him to see me like that.’
Impact on daily life: relationships, work, finances and recreational activities	
Positive impact on relationships	‘One really positive thing to come out of it is to be much more upfront and open’, ‘So it has allowed me, it's given me the balls and the confidence to be like “I'm not handling things very well today, I really need a bit of space” or “I need a bit more help with this” so that's really positive I think.’
Negative impact on relationships	‘I mean it's very, very difficult, knowing the stress I've put on my nearest and dearest. Not through any fault of my own but I know it was hugely traumatic for a lot of people I really, really love and that was quite difficult.’
Impact on social life	‘It's not too bad with people who I know, know very well, they understand but you know, meeting new people to do that, it's, it's awkward.’
Impact on hobbies	‘I simply can't anymore. It's no good, I have handed in our golf equipment, which is the saddest part about it, I can say. Consequently, I cannot walk that far.’
Impact on holidays/travel	‘It's always difficult for me now getting insured to go, getting insured to go somewhere like New York would just be an impossibility. Er, just in case, the worst came to the worst, and I needed to be admitted into hospital anywhere. I couldn't get covered for existing illnesses.’
Financial impact	‘I pay about between $600–650 per month for my pouches and gadgets that I need for my colostomy.’
Impact on work or career	‘I had no choice, I had to stop work. I had no choice.’ ‘Well, to be honest, it probably helped because it gave me something to focus on rather than the illness itself at the time.’
Retirement	‘I felt very disappointed at first because you know, retiring, I'd worked so hard. I worked as a nurse for 43 years and you know, you feel a bit angry and disappointed that as soon as you retire, everybody dreams of being able to travel and do all sorts of things and I just ended up as a patient.’
Managing at home	‘Because I can't stand for very long to do any washing up or any cooking. So, I've got carers to come and do the cooking and the cleaning.’
Feelings surrounding life now, adapting and the future	
Life returning to normal or a ‘new normal’	‘Well, I've tried to conduct my life as normal as possible because I fear that I nearly lost it during the second operation and obviously I was in hospital for about 9–10 months which was really unpleasant for me and my family.’
Acceptance	‘There's nothing about my … everything else has been a challenge, you know, what I've had to work through at work, it just affects everything, everything else, so the only way that it can be positive is through acceptance.’
Gratitude	‘I live life more intensely now. I sometimes tell my partner that even if I were to die tomorrow, the years I've had since, my “second chance” if you like, were lived so much more intensely than my life prior to that. It never would have happened without this disease, not in 80 years of living. So I'm really lucky in that sense.’
Managing one's own health	‘Erm, well, I was in a wheelchair for quite some time and it's only through sheer determination that I managed to put one foot in front of the other.’
Positive feelings about the future	‘I have a bright outlook on the future [laughs]. No, I mean I have really, a really bright outlook on the future. I'm kind of so happy with where I am at and how I feel.’
Negative feelings about the future	‘You've got an uncertain future, haven't you? You can never plan too far ahead because you don't know what the future holds.’
The impact of coexisting long‐term health conditions	‘I know in recent years, I haven't really been on holidays abroad or anything, you see, my major problem with me is my COPD [chronic obstructive pulmonary disease]. That's gotten worse and worse, of course that does affect you because your breathing becomes very difficult. You know, where I didn't know or suffer any real pain with the cancer but I'm suffering with the COPD.’
Effect of ageing	‘Of course, it's not like it was before the surgery, but I'm having trouble determining whether it's due to the cancer or my age. I don't know it as well as I used to, but I've also gotten older.’

**TABLE 3:** Feelings on adjusting to life following diagnosis and during treatment theme with illustrative quotations.

Reflections on adjusting to life following diagnosis and during treatment	
Positive feelings surrounding diagnosis and treatment	‘Even though, the surgery was far more complicated and had a lot more sort of, after effects, symptoms. I suppose like childbirth, the second time round is just a bit less terrifying.’
Negative feelings surrounding diagnosis and treatment	‘Yes, what it was like to have a relapse, it was quite traumatic’, ‘So then I sort of had a sort of uh, panic attack almost but it passed quickly’
Decisional regret and other feelings surrounding the decision to have surgery and other treatments	‘Like if I had to choose between knowing this would happen to me with radiation and risking having to have a permanent colostomy, I would have chose permanent ostomy without ever doing the radiation.’

#### Experiences of long‐term follow‐up care

All participants had received their care for LRRC at specialist centres, often geographically distant from their home. Some expressed their willingness to travel for follow‐up, whereas others reported the negative impact on their time and finances. Feelings regarding the end of follow‐up were mixed: some felt positively, with a sense of relief, whereas others would prefer to continue having follow‐up, particularly scans to monitor for recurrence as this provided reassurance. Several participants had required further interventions or procedures to manage complications of treatment, such as surgery for bowel obstruction or parastomal hernia. Participants reported various sources of support, including healthcare professionals, family, employers, support groups and others with similar experiences.

#### Unmet needs and areas for improvement

Participants identified several unmet needs within their treatment or follow‐up, including information regarding nutrition and diet, stoma management and discussion regarding sexual function. Some female participants particularly felt that the emotional impact of impaired sexual function was not addressed. Aspects of care that could be improved included involving patients more in decisions, better communication between different hospitals and clinical teams, earlier recognition and diagnosis of treatment‐induced menopause in younger female patients and better access to high‐quality MRI scanning at peripheral hospitals.

#### Long‐term physical effects of cancer and treatment

Long‐term physical effects due to LRRC and its treatment included problems related to wounds: parastomal and perineal hernias, perineal sinuses or fistulas and associated symptoms such as pain and discharge. Chronic pain included pain in the buttocks, perineum or rectum, pain related to the sciatic nerve and pain in the groin or abdomen. A range of issues related to mobility were identified, including leg weakness, swelling, stiffness, foot drop and peripheral neuropathy. Urinary symptoms included incontinence and voiding difficulties. Other long‐term effects included altered bowel function, vaginal bleeding, treatment‐induced menopause and impaired fertility. Generalized symptoms included fatigue and oral mucosal problems.

#### Living with a stoma, urostomy or other urinary device

Stoma‐related problems were a common issue, and included bleeding from the stoma, bag leaks, skin excoriation, high stoma output and difficulty maintaining a seal in hot weather. The ways in which a stoma could impact on daily life included avoiding travelling and social activities far from toilet facilities and refraining from romantic relationships or sexual activity. Some participants described the difficulty they had experienced in accepting their stoma initially, though many felt more positive over time. Participants who had experienced poor preoperative bowel function found their stoma a vast improvement. Some participants had a urostomy and others required urinary devices, including suprapubic catheter, ureteric stent and nephrostomy, describing challenges including recurrent infections. Positive experiences regarding urostomy included not needing to wake at night to urinate.

#### Long‐term psychological impact

Several participants experienced negative body image, feeling conscious of stomas, hernias and scars to their abdomen and perineum. This affected confidence in relation to their social life and romantic relationships. Anxiety, particularly in relation to scans and the fear of re‐recurrence, was described. Long‐term symptoms such as chronic pain and functional limitations had a negative impact on mental health. Participants experienced low mood, feeling a lack of control and isolation. Feelings surrounding returning to ‘normal’ life were complex, including a sense of grief for one's former self. Participants also identified positive effects and coping mechanisms, including resilience, positive attitude, a greater appreciation for life and strengthening of their existing faith.

#### Impact on sexual function and intimate relationships

Several participants experienced reduced sexual interest. Most male participants experienced erectile dysfunction and female participants described vaginal discomfort or pain during sexual activity, inability to partake in penetrative intercourse due to vaginal atrophy and impaired sensation in the vulva and clitoris. Urinary leakage or the need for a urinary catheter also negatively affected sexuality. Some participants had accepted this, or had adapted using medication for erectile dysfunction or exploring intimacy without penetration. Other participants had found their inability to have penetrative sexual intercourse much more difficult from both an emotional and physical perspective. Negative body image also affected confidence during sexual intercourse. For others, however, impaired sexual function was not an issue as they were no longer sexually active for other reasons.

#### Impact on daily life: relationships, work, finances and recreational activities

Participants highlighted the emotional and psychological impact of LRRC on their families. Some participants described strengthening of their relationships, others had lost touch with friends or experienced a breakdown in their relationship with their partner. Recreational activities, such as cycling or golf, were no longer possible for many participants, others described being able to continue with hobbies, particularly less physically demanding ones. Some participants avoided travelling due to their stoma. Financial implications included needing to pay for stoma supplies. For some, returning to work following LRRC treatment was not an option, others described continuing to work during treatment and finding this a helpful distraction. Some participants needed support from carers to manage at home.

#### Feelings surrounding life now, adapting and the future

For some participants, life had returned to how it had been before surgery, others described striving to maintain a sense of normality following treatment. Some described a sense of not being able to return to life as it had been before. Many participants described a process of acceptance in relation to the lasting impact of LRRC and its treatment in lieu of the alternative. Despite living with considerable consequences, most participants described being satisfied with their lives, and some attributed this to adaptation. Gratitude was expressed by several participants, including the feeling of being given a ‘second chance’. In relation to the future, many participants were ‘taking each day at a time’. Some felt confident that they could face what the future holds, having come through their experience of LRRC. Some participants worried about how they would manage with increasing age, for example their ability to handle a stoma independently, others described feeling generally uncertain about the future.

#### Reflections on adjusting to life following diagnosis and during treatment

Participants described feeling satisfied with their outcome, particularly given that, for the majority, extensive surgery represented the only possibility of cure. Experiences of diagnosis and treatment were described as traumatic by many participants, while some felt it was easier to handle the recurrence than the primary tumour. The process of recovery was described as long and challenging, with participants initially in ‘survival mode’ before starting to process their experience and deal with the lasting effects. Some participants expressed regret in relation to aspects of their treatment, this included regretting the decision to have radiotherapy given their subsequent experience of vaginal atrophy or removing the ovaries resulting in early menopause.

## DISCUSSION AND CONCLUSIONS

The eight major survivorship themes identified in this study demonstrate the enduring impact of LRRC and its treatment, establishing that longer‐term survivors of LRRC continue to experience similar issues to those previously described by patients up to 2 years following diagnosis or treatment [21, 23]. These previously identified issues include symptoms such as pain, lethargy, gynaecological, gastrointestinal and urological symptoms, and mobility issues including side effects of surgery [21, 23], many of which were found to continue into longer‐term survivorship. Other previously described issues, including sexual function, social functioning and role functioning [23], were also found to be relevant to longer‐term survivors. The lasting psychological impact and future perspectives following LRRC and its treatment also show similarities with issues previously described, including a desire to return to ‘normality’ and feelings of helplessness or loss of identity [21, 23]. Existing evidence regarding long‐term survivorship following LRRC is limited, therefore documenting and improving understanding of the lived experiences of this patient group represents an important step in advancing this field of work. Overall, both positive and negative experiences were described across a wide range of domains. Despite detailing the pervasive and sometimes burdensome long‐term impact of LRRC and its treatment, participants were generally accepting of their ‘new normal’ and had adapted well.

The similarities in experiences described by participants in this study, who were a median of 5 years posttreatment, to those previously described in patients closer to diagnosis is a notable finding in the context of existing cancer survivorship literature. The growing body of evidence regarding survivorship in primary malignancies largely reports longer‐term survivorship issues that are different from those experienced by patients during treatment [20]. This study highlights the importance of understanding disease‐specific survivorship, particularly in LRRC, given the complex nature of both the disease and its management, to gain a better understanding of patient experiences. The apparent discrepancy in the wide range of survivorship issues the study participants continued to experience and the high levels of overall satisfaction reported by the majority could be attributed to response shift [29]. Response shift describes a process of individuals adjusting to and accepting their new situation over time, thus experiencing better HRQoL, although their symptoms and objective situation remain unchanged or have deteriorated. Accordingly, acceptance was identified as a subtheme in this study, and participants also described experiencing gratitude and a renewed appreciation for life.

Long‐term survivorship care following treatment for advanced pelvic malignancy, including LRRC, is likely to represent an important area of interest as the number of survivors continues to rise. To our knowledge there have been no published descriptions of dedicated survivorship care interventions or clinics for this specific group of patients. However, there are numerous potential approaches that could be applied to address the unmet needs and issues highlighted in this study. Support regarding sexual function could be improved through the introduction of routine access to sexual health practitioners or counselling within standard LRRC follow‐up care [30, 31]. Previously identified barriers to the delivery of aftercare regarding sexual function include clinicians lacking confidence in discussing sexual function [32, 33, 34] or assuming a lack of relevance based on characteristics such as age [32]. Training could be offered to clinicians to facilitate high‐quality delivery of this important aspect of survivorship care [35, 36]. Where communication is concerned, several participants identified their dedicated specialist nurse as a significant source of support during their treatment, follow‐up care and beyond. Ensuring all patients with LRRC have access to a dedicated specialist nurse may help them to feel more supported in navigating their treatment and follow‐up pathways. Other options could include access to virtual survivorship care interventions [37], which could particularly benefit those living far from their treating centre. In relation to delivering improvements in survivorship care, the ACPGBI IMPACT study is currently under way [38] and will help to more clearly define the issues that need to be addressed in current care pathways for patients with advanced colorectal cancer. Most importantly, the development of any targeted survivorship interventions should be undertaken with input from patients and other key stakeholders.

There are several strengths to this study, including the robust qualitative methodological approach employed, utilizing a framework method for thematic analysis. Selection of this approach was carefully considered and felt to be best suited to the aims of the project and plans for collaborative, international working. Furthermore, all interview facilitators had either received training in qualitative methods or were experienced qualitative researchers. The major strength of the study was the multicentre, international approach to recruitment, with a view to demonstrating that the long‐term impact of LRRC and its treatment is apparent across different cultural determinants. Sites were not included in the purposive sampling strategy to allow for an inclusive group of patients from all centres performing exenterative surgery. The approach to international recruitment and analysis was carefully planned with close collaborative working to ensure that conceptual equivalence was maintained and not lost in translation. Additionally, the multiple approaches for interviewing participants, including in person, via telephone and via videoconference, widened participation. One of the major limitations of the study is the use of the LRRC‐QoL conceptual framework to inform the interview topic guide, which may have influenced the themes identified. However, the two main interviewer facilitators (NM and SW) did not perceive this to significantly influence the content of the interviews, as most of the conversation was generated by open‐ended questions that did not relate to the LRRC‐QoL. There may also have been self‐selection bias in the study participants, as patients with extremes of opinions or experiences may be more likely to respond [39, 40, 41]. Other limitations include the lack of diversity in the participants recruited, with the majority being of White ethnicity (*n* = 16, 61.5%) or English speaking (*n* = 17, 65.4%), and all were recruited from developed countries.

In conclusion, the wide range of survivorship issues identified in this study reflect the enduring impact of LRRC and the complexity of its management. There are several unmet needs that could be addressed to improve patient care; however, most participants had adapted well over time and described being satisfied with their lives overall, despite the consequences of LRRC and its treatment. It is important to acknowledge that exenterative surgery generally represents the only curative treatment option for patients with LRRC, which is likely to factor in them accepting its enduring effects.

## AUTHOR CONTRIBUTIONS

**Niamh McKigney:** Conceptualization; writing – original draft; project administration; data curation; formal analysis; methodology; investigation. **Sophia Waldenstedt:** Formal analysis; writing – original draft; resources; data curation; investigation; conceptualization. **Elisabeth Gonzalez:** Formal analysis; resources; investigation; writing – review and editing. **Jan M. van Rees:** Resources; investigation; writing – review and editing. **Henriette Vind Thaysen:** Investigation; resources; writing – review and editing. **Eva Angenete:** Writing – review and editing; supervision; investigation; conceptualization. **Galina Velikova:** Conceptualization; writing – review and editing; supervision; methodology. **Julia M. Brown:** Supervision; writing – review and editing; conceptualization; methodology. **Deena P. Harji:** Funding acquisition; writing – review and editing; supervision; methodology; conceptualization; formal analysis; project administration. **Henrik Kidmose Christensen:** Resources. **Joost Rothbarth:** Resources. **Cornelis Verhoef:** Resources. **Elliott Gee:** Resources. **Kaitlyn Ulmer:** Resources. **Ahmer Karimuddin:** Resources. **Claire Taylor:** Resources. **Laura Gould:** Resources. **Elaine Burns:** Resources. **John T. Jenkins:** Resources. **Chloe Hardy:** Resources. **Christopher Mann:** Resources. **Kirsten Boyle:** Resources. **David McArthur:** Resources. **Mit Dattani:** Resources. **Cath Moriarty:** Resources. **Peter Sagar:** Resources. **Tamara Glyn:** Resources. **Frank Frizelle:** Resources. **Helen Mohan:** Resources. **Satish Warrier:** Resources. **Alexander Heriot:** Resources. **Tracy Fitzsimmons:** Resources. **Tarik Sammour:** Resources.

## FUNDING INFORMATION

Funding for the study was provided by Bowel Research UK, Pelican Cancer Foundation, the agreement concerning research and education of doctors ALFGBG‐965084, the Swedish Cancer Society 22 2265 Pj and Assar Gabrielssons foundation FB21‐117, and Anna‐Lisa and Bror Bjornssons foundation.

## CONFLICT OF INTEREST STATEMENT

The authors have no conflict of interest to declare.

## ETHICAL APPROVAL

The study was approved by the West of Scotland Research Ethics Committee 3 on 8 October 2020 (ref. 20/WS/0116) with additional local ethical approvals at each participating international centre. All participants provided consent.

## Supporting information


Figure S1.

## Data Availability

The data that support the findings of this study are available on request from the corresponding author. The data are not publicly available due to privacy or ethical restrictions.

## APPENDIX A

LRRC‐QoL collaborators: Henrik Kidmose Christensen (Department of Surgery, Aarhus University Hospital, Aarhus, Denmark), Joost Rothbarth, Cornelis Verhoef (Department of Surgical Oncology and Gastrointestinal Surgery, Erasmus MC Cancer Institute, University Hospital Rotterdam, The Netherlands), Elliott Gee, Kaitlyn Ulmer, Ahmer Karimuddin (Department of Surgery, St Paul's Hospital, University of British Columbia, Vancouver, BC, Canada), Claire Taylor, Laura Gould, Elaine Burns, John T. Jenkins (St Mark's Hospital, London North West University Healthcare NHS Trust, Harrow, UK), Chloe Hardy, Christopher Mann, Kirsten Boyle (Leicester Royal Infirmary, University Hospitals of Leicester, Leicester, UK), David McArthur, Mit Dattani (Heartlands Hospital, University Hospitals Birmingham NHS Foundation Trust, Birmingham, UK), Cath Moriarty, Peter Sagar (John Goligher Colorectal Surgery Unit, St James's University Hospital, The Leeds Teaching Hospitals NHS Trust, Leeds, UK), Tamara Glyn, Frank Frizelle (The Department of Surgery and Critical Care, University of Otago, Christchurch, New Zealand; Department of Surgery, Te Whatu Ora Health New Zealand Waitaha Canterbury, Christchurch, New Zealand), Helen Mohan, Satish Warrier, Alexander Heriot (Department of Cancer Surgery, Peter MacCallum Cancer Centre, Melbourne, Australia), Tracy Fitzsimmons (Colorectal Unit, Department of Surgery, Royal Adelaide Hospital, Adelaide, Australia; Department of Surgical Specialties, Faculty of Health and Medical Sciences, The University of Adelaide, Adelaide, Australia), Tarik Sammour (Colorectal Unit, Department of Surgery, Royal Adelaide Hospital, Adelaide, Australia).
