# Supplementary material for: Survivorship issues in long‐term survivors of locally recurrent rectal cancer: A qualitative study
Source: Colorectal Dis. 2025 Mar 20;27(3):e70051. doi: 10.1111/codi.70051 (PMC11923726; doi:10.1111/codi.70051)
Supplement: Supplementary file 1 — Figure S1. [file CODI-27-0-s001.docx]

Supplementary Material

Figure S1: Purposive Sampling Strategy

**Researcher Reflective Log**

Overall, I found facilitating the interviews to be a real privilege as it allowed me to gain a better understanding of the personal experiences of patients in a way which is difficult to gather during clinical encounters given the time constraints. My background as a clinician may have made patients feel more comfortable speaking to me about their experiences given a degree of professional trust. I suspect that my gender may have also put some patients, particularly female patients, at ease in relation to discussing sensitive topics such as sexual function. Conversely, my gender and age may have been a barrier to some patients. Participants may also have found it difficult to share negative healthcare experiences due to me being a ‘member of the establishment’. Other researchers involved in conducting interviews internationally (JvR and SW) also reported feeling privileged to have heard the experiences of these patients during the in-depth interviews.

My clinical background and knowledge of the themes which arose from the development of the LRRC-QoL, is likely to have influenced the analysis and the lens through which I coded the data, informed by the bio-psycho-social approach I had learned at medical school and my own experiences from clinical practice. I tried to use this to my advantage to inform the analysis, whilst also ensuring that the themes identified reflected the experiences and feelings of the patients interviewed. I did this by returning to the transcripts frequently to ensure they were represented in the analytic framework.
